# Comparative Evaluation of Propofol, Eugenol and MS-222 in Yellowtail Tetra *Astyanax lacustris*: Anesthetic Efficacy, Physiological Responses and Recovery Dynamics

**DOI:** 10.3390/ani16213474

**Published:** 2026-11-03

**Authors:** Bianca da Costa Tavares da Silva, Leonila Ester Reinert Raspantini, Silvana Lima Górniak

**Affiliations:** Department of Veterinary Pathology, School of Veterinary Medicine and Animal Sciences, Research Center for Veterinary Toxicology (CEPTOX), University of São Paulo, São Paulo 13635-900, Brazilgorniak@usp.br (S.L.G.)

**Keywords:** anesthetic protocols, aquaculture, fish anesthesia, tropical fish, welfare

## Abstract

In both aquaculture and scientific experimentation, fish handling often requires anesthesia to facilitate immobilization and minimize handling-associated disturbances. This study aimed to compare the anesthetic performance of different anesthetic agents in *Astyanax lacustris*, assessing induction, recovery, and safety to support protocol selection for tropical freshwater fish. A total of 114 yellowtail tetra *Astyanax lacustris* were used, including 54 fish in a preliminary experiment and 60 fish in the main experiment, with 10 fish per concentration in each anesthetic treatment during the main experiment. Fish were exposed to different concentrations of propofol (5.0 and 6.5 mg L^−1^), eugenol (40 and 50 mg L^−1^), and MS-222 (120 and 130 mg L^−1^). Anesthetic efficacy and complementary physiological responses were assessed through induction and recovery times, responsiveness during recovery, opercular beat rate (OBR), hematological parameters, and plasma concentrations of glucose, lactate, and cortisol. Data were analyzed using analysis of covariance (ANCOVA) followed by appropriate post hoc tests (*p* < 0.05). All anesthetics successfully induced stage IV anesthesia at the evaluated concentrations, although distinct induction and recovery profiles were observed. MS-222 promoted significantly faster progression to deep anesthesia and shorter recovery times, whereas propofol induced prolonged recovery and marked reduction in OBR, with residual ventilatory depression after recovery. Eugenol showed an intermediate anesthetic profile associated with pronounced excitation during induction and an altered recovery pattern. Hematological parameters and plasma lactate concentrations did not differ significantly among anesthetic types or concentrations. Plasma glucose concentrations increased at higher anesthetic concentrations, whereas plasma cortisol concentrations differed significantly between propofol and eugenol groups (*p* < 0.05). Overall, the findings demonstrate that propofol, eugenol, and MS-222 produce distinct anesthetic and ventilatory responses in *A. lacustris*, highlighting the importance of selecting anesthetic protocols according to the intended experimental or handling procedure.

## Simple Summary

Fish anesthesia is widely used in aquaculture and scientific research to reduce handling stress, improve animal welfare, and facilitate routine procedures. However, different anesthetics can produce distinct physiological effects, making it important to identify protocols that are both effective and safe for each species. In this study, we compared the anesthetics propofol, eugenol, and MS-222 in the yellowtail tetra (*Astyanax lacustris*), an emerging Neotropical model fish. Although all three agents successfully induced deep anesthesia, they differed in induction and recovery characteristics, respiratory effects, and stress-related responses. These findings provide practical information for selecting the most appropriate anesthetic according to the intended procedure, contributing to improved fish welfare and more reliable experimental and aquaculture practices.

## 1. Introduction

Anesthetic agents are widely employed during fish handling to facilitate manipulation procedures and minimize handling-associated disturbances. Procedures such as transport, biometrics, blood sampling, vaccination and tagging may induce neuroendocrine metabolic pathways, leading to alterations in cortisol release, glucose metabolism and ventilatory alterations, potentially affecting animal welfare, recovery quality and experimental consistency [1,2]. Consequently, the selection of appropriate anesthetic protocols is important not only for safe handling and immobilization, but also for promoting adequate recovery and reducing procedure-associated physiological disturbances in fish.

Given its practicality and applicability under aquaculture conditions, anesthesia in fish is commonly performed by immersion. In this approach, the anesthetic agent is diluted in water within a separate tank, allowing drug absorption primarily through the gills and, to a lesser extent, through the skin. This method enables the simultaneous induction of multiple individuals while reducing handling-associated disturbances [3]. Under these conditions, different anesthetic agents may be used, and their selection depends on factors such as availability, cost, species-specific responses and the intended purpose of anesthesia.

In general, tricaine methanesulfonate (MS-222) remains one of the most widely used anesthetics in fish and is currently the only compound approved by the U.S. Food and Drug Administration (FDA) for use in food fish, with established withdrawal periods [4]. For tropical freshwater fish species, however, eugenol (the main component of clove oil) has become a commonly used alternative due to its high efficacy and lower cost when compared to MS-222 [5]. In research and aquaculture contexts, other anesthetic agents, such as benzocaine, 2-phenoxyethanol, quinaldine and propofol, have also been employed [6]. Among these agents, propofol has attracted increasing interest for experimental application in fish species because of its anesthetic efficacy and widespread clinical use in veterinary and human medicine [7].

Many studies have investigated the advantages and disadvantages of different anesthetic agents used in fish [8,9,10]. However, a substantial proportion of this knowledge has been generated from studies in temperate or cold-water species, particularly salmonids, whereas information on small tropical freshwater and Neotropical fish species remains comparatively limited [10,11]. In addition to species-specific physiological characteristics, water temperature represents an important factor influencing anesthetic performance in fish, as it directly affects metabolic rate, ventilatory activity, and the pharmacokinetics of anesthetic agents. Consequently, temperature-dependent variations may significantly impact both the efficacy and safety of these compounds [12,13].

Yellowtail tetra *Astyanax lacustris* (Lütken 1875) was selected for the present study because it represents a well-established tropical fish research model, with favorable biological characteristics for experimental research, including small size, high reproductive capacity, and robustness [14,15,16].

Three anesthetic agents were selected for evaluation: eugenol, propofol and MS-222, based on their widespread use and distinct pharmacological properties in tropical, temperate and cold-water fish species [3,9,17,18]. These anesthetic agents possess distinct mechanisms of action, allowing comparative assessment of distinct anesthetic response profiles. Eugenol modulates ion channels and enhances inhibitory neurotransmission in the central nervous system. Propofol acts primarily through potentiation of GABAA receptor–mediated inhibitory neurotransmission and is widely employed in veterinary and human medicine [19]. In contrast, MS-222 exerts its anesthetic effects through blockade of voltage-gated sodium channels, inhibiting neuronal depolarization and impulse conduction [20].

To evaluate the anesthetic performance of these agents, this study assessed induction and recovery responses, ventilatory activity through opercular beat rate (OBR) and complementary hematological and biochemical parameters following anesthetic exposure. The evaluated parameters were selected to provide complementary information regarding physiological responses associated with anesthesia and handling procedures in *A. lacustris*. In addition, induction and recovery characteristics together with ventilatory activity represent important indicators of anesthetic efficacy and recovery quality in fish [21,22,23]. Although previous studies have investigated the anesthetic efficacy and physiological responses to these agents in *A. lacustris* [8,24], none have directly compared the three anesthetics under the same experimental conditions while simultaneously evaluating physiological parameters and induction and recovery dynamics to determine the most appropriate concentrations.

Given the limited information available for tropical freshwater species and the distinct pharmacological mechanisms of these anesthetic agents, we hypothesized that eugenol, propofol, and MS-222 would produce different induction and recovery profiles as well as distinct ventilatory and anesthetic response patterns in *A. lacustris*. Accordingly, this study aimed to compare their anesthetic performance by assessing variables relevant to protocol selection, providing evidence for the development of appropriate anesthetic protocols for tropical freshwater fish.

## 2. Materials and Methods

### 2.1. Fish and Experimental Facilities

A total of 114 *A. lacustris* (*n* = 114) were used in this study, with 54 fish allocated to the preliminary experiment (*n* = 18 per anesthetic; *n* = 6 per concentration) and 60 fish to the main experiment (*n* = 20 per anesthetic; *n* = 10 per concentration). Fish were equally distributed by sex, weighed 15 ± 5 g, measured 7–12 cm in length, and were fed a commercial diet (Guabitech Aqua^®^ 1.7 mm, Guabi Nutrição e Saúde Animal, Sales de Oliveira, SP, Brazil) twice daily at 2% of body weight per day. Before anesthetic exposure, all animals were fasted for 12 h. The fish were housed in three recirculating systems composed of 0.2 m^3^ tanks at the Research Center for Veterinary Toxicology (CEPTOX), University of São Paulo (USP), Pirassununga, Brazil (21°58′ S, 47°27′ W). The system had mechanical, biological and ultraviolet filters and experimental conditions were kept under continuous aeration (dissolved oxygen 6.0 ± 0.3 mg L^−1^), 12:12 h light/dark photoperiod and constant temperature control (28 ± 0.3 °C). Water quality parameters were monitored periodically, showing a conductivity of 600 ± 50 S/cm, salinity of 0.3 ± 0.05 ppt, and total dissolved solids (TDS) of 300 ± 25 ppm.

For the anesthetic assays, fish were individually transferred from the holding systems to 2 L aquaria containing the anesthetic solution for induction evaluation and subsequently transferred to separate 2 L aquaria containing system water for recovery assessment. Fish were equally sampled from all aquaria and recirculating systems to minimize potential sampling bias. Afterwards, fish were transferred back to the recirculating systems.

### 2.2. Chemicals

Reference standards of eugenol (≥99% purity) and tricaine methanesulfonate (MS-222 ≥ 98% purity), both of analytical grade, were purchased from Sigma-Aldrich (São Paulo, Brazil). Propofol was used as a commercial formulation (10 mg mL^−1^ lipid emulsion; Propovan, Cristália, São Paulo, Brazil), which enables its direct dilution in water for preparation of the anesthetic baths [25,26]. Anesthetic solutions were freshly prepared with stock solutions of each chemical in system water, with the exception of propofol, administered in its original commercial formulation.

### 2.3. Anesthetic Solutions

Eugenol stock solution was prepared in 96% ethanol (Dinâmica Química Contemporânea, Indaiatuba, SP,, Brazil) at a 1:9 ratio (*v*/*v*; eugenol:ethanol), resulting in a final stock concentration of 106.6 mg mL^−1^, according to previously established protocols [27,28]. The final ethanol concentrations in the immersion baths ranged from 0.034% to 0.042% (*v*/*v*), depending on the anesthetic concentration used. MS-222 stock solution was prepared at 10 g L^−1^ in deionized water [29]. MS-222 final anesthetic solution was buffered with sodium bicarbonate (Dinâmica Química Contemporânea, Indaiatuba, SP, Brazil) until reaching pH 7.0, which was monitored using a portable pH meter (AKSO, model AK90, AKSO, São Leopoldo, RS, Brazil). The immersion baths were prepared in 2 L fiberglass tanks with a final working volume of 1 L and each fish was anesthetized individually.

### 2.4. Anesthetic Profile and Determination of Experimental Concentrations

To determine two optimal concentrations for *A. lacustris*, a preliminary study was done using 18 fish per anesthetic treatment, totaling 54 individuals out of the 114 initially allocated. Three concentrations of each anesthetic were tested in six animals per concentration (three males and three females): eugenol (30, 40, 50 mg L^−1^), MS-222 (90, 120, 150 mg L^−1^) and propofol (5.0, 6.5, 7.5 mg L^−1^). The selected concentrations were based on previous studies in *A. lacustris* and other tropical species, such as Nile tilapia *Oreochromis niloticus* [9,28,30,31].

The two selected concentrations in the preliminary phase were applied in the main experiment, which was conducted using the remaining 60 individuals from the initial sample. In this phase, fish were allocated in groups of 10 individuals (five males and five females) per treatment. These concentrations had to meet the criteria of inducing within 3 min and allowing recovery of equilibrium within 5 min [32,33]. For this experiment, the fish were kept in immersion bath until stage IV of anesthesia, determined by loss of equilibrium and loss of painful stimuli [33,34] and then were relocated to another 2 L glass tank with system water.

Both the preliminary and main experiments included behavioral assessments, for which opercular beat rate (OBR; beats min^−1^) was measured before anesthesia, at loss of equilibrium (stage III anesthesia), at loss of response to tail-pinch (stage IV anesthesia), and after recovery. The times to loss of equilibrium (T1) and loss of the tail-pinch reflex (T2), as well as the times to regain movement (T3), equilibrium (T4) and complete recovery (T5) were also recorded [35,36]. To ensure consistency in the assessment of these parameters, their determination was based on the consensus of three trained observers.

### 2.5. Hematological Analysis

Blood sampling was conducted exclusively in the main experiment, involving the 60 individuals from the initial sample, since stage III anesthesia is considered the most appropriate level for blood collection, as opposed to stage IV, used in the anesthetic performance assessments [37].

Seven hematological parameters were evaluated after anesthesia: hemoglobin concentration (Hb), hematocrit level (HCT), mean corpuscular volume (MCV), mean corpuscular hemoglobin content (MCH), mean corpuscular hemoglobin concentration (MCHC), red blood cell counts (RBC), white blood cell counts (WBC) and differential leukocyte counts (lymphocytes, neutrophils, monocytes and eosinophils).

Upon reaching stage III of anesthesia, the fish were removed from the anesthetic bath and placed on a wet cloth for blood collection. Blood samples were obtained from the caudal vein using a heparinized 1 mL syringe and placed in a 0.5 mL microcentrifuge tube. Afterwards, a 10 μL hematocrit tube of each sample was filled with blood and centrifuged for 5 min at 12,000× *g*. HCT was measured using the microhematocrit method [38].

Erythrocyte (RBC) and leukocyte (WBC) counts were determined by manual counting using a Neubauer hemocytometer with Natt–Herrick’s solution as a diluent dye at a dilution of 1:200 [38], and hemoglobin (Hb) concentration was measured spectrophotometrically at 540 nm [39]. Blood smears were prepared and stained with May-Grünwald-Giemsa (MGG) to obtain differential leukocyte profiles [40]. RBC, MCV, MCH, and MCHC were calculated from RBC, HCT, and Hb using standard formulas.

$$\mathrm{MCV}=HCT\times \frac{10}{RBC}$$

$$\mathrm{MCH}=Hb\times \frac{10}{RBC}$$

$$\mathrm{MCHC}=Hb\times \frac{100}{HCT}$$

After blood samples were collected for hematological analysis, the remaining blood was centrifuged at 3000× *g* for 15 min, and plasma was aspirated from the supernatant for glucose, lactate, and cortisol measurements. Plasma glucose and lactate concentrations were determined in all animals using commercial biochemical assay kits (Labtest, Lagoa Santa, Minas Gerais, Brazil).

Cortisol levels were quantified by radioimmunoassay (RIA) [41] in 40 animals, equally distributed between groups, since adequate plasma volume could not be obtained from all 60 individuals. For each anesthetic and concentration, three males and three females were analyzed. In addition, three extra males from the lower concentration of each anesthetic and one extra male from the higher concentration of propofol were included due to sample availability. For this analysis, a commercial Cortisol CT kit (MP Biomedicals, LLC, Solon, OH, USA) was used. Antibody cross-reactivity with cortisol was 100% and the lower detection limit of the assay was 0.17 μg/dL. The intra- and inter-assay coefficients of variation (CV) were <9.3% and <10.2%, respectively.

### 2.6. Statistical Analysis

Statistical analyses were performed exclusively on the main experiment (*n* = 60, with a subset of *n* = 40 for cortisol). Data were expressed as means ± standard error of the mean (SEM). A full-factorial three-way Analysis of Covariance (ANCOVA) was used to evaluate the effects of anesthetic type, concentration, and sex (fixed factors), as well as their two-way and three-way interactions, on induction and recovery times, as well as on biochemical and hematological parameters, using fish body weight as a covariate to adjust for potential weight-dependent effects. For OBR, repeated measures ANOVA was performed to evaluate changes across the experimental stages, since this variable was repeatedly measured in the same individuals over time. A separate one-way ANOVA was performed to compare baseline OBR values among experimental groups, confirming the absence of significant differences prior to anesthetic exposure. Assumptions of normality, homoscedasticity and homogeneity were verified across all analyses using Shapiro–Wilk, Levene’s tests and factor x covariate interaction terms. When necessary, data were log-transformed to meet these assumptions. Regarding cortisol, ANCOVA models were computed using Type III Sum of Squares to account for minor sex imbalances due to plasma availability (n=40). Additionally, a secondary ANCOVA was performed on a strictly balanced subset of 36 fish (18 males and 18 females; 3 males and 3 females per group) to ensure the primary analysis was free of bias. When significant main effects were found (*p* < 0.05), Tukey’s post hoc test was used for multiple comparisons. All statistical analyses and graphical representations were performed using Jamovi (version 2.6) and Microsoft Excel.

## 3. Results

### 3.1. Preliminary Study

The selected concentrations of each anesthetic were determined based on pre-established criteria, including the absence of adverse events, such as abnormal behavior, respiratory arrest or mortality, during induction up to stage IV anesthesia and subsequent recovery. Additionally, fish were required to reach stage III of anesthesia within 3 min and to recover equilibrium within 5 min. These criteria were established considering that the main experiment will involve blood sampling, an invasive procedure that may increase the risk of hypovolemia in such small animals. Therefore, the occurrence of adverse effects even in the absence of additional experimental stressors would indicate an increased risk of mortality during procedures involving blood collection.

Based on these criteria, 30 mg L^−1^ eugenol, 150 mg L^−1^ MS-222, and 7.5 mg L^−1^ propofol were excluded from the experiment, as respiratory arrest requiring manual ventilation for recovery was observed in one to two animals within each treatment concentration. In the 90 mg L^−1^ MS-222 treatment, 50% of the tested animals exhibited muscular spasms between stages III and IV of anesthesia, with persistent involuntary movements even after loss of the tail-pinch reflex. The concentrations selected for the main experiment were chosen to represent an effective range while avoiding the adverse responses observed during the preliminary assessment. As such, the lowest and highest effective concentrations selected for the main experiment were 5.0 and 6.5 mg L^−1^ for propofol, 120 and 130 mg L^−1^ for MS-222, and 40 and 50 mg L^−1^ for eugenol. Although 130 mg L^−1^ MS-222 was not directly evaluated in the preliminary trial, it was selected as an intermediate concentration between 120 and 150 mg L^−1^ to further investigate the anesthetic response within the effective range.

### 3.2. Anesthetic Profile

At all tested concentrations of each drug, fish reached all four stages of induction and recovery from anesthesia. No mortality was observed with any of the agents. Behavioral responses were evaluated through both qualitative assessment and statistical analysis. Qualitatively, the effects were similar within each anesthetic, with concentration only modulating the magnitude of the response.

During recovery from propofol, 35% of the fish (7/20) exhibited stereotyped swimming associated with unresponsiveness to external stimuli, including tactile stimulation and obstacle avoidance. This altered recovery pattern was predominantly observed at the lowest propofol concentration (5.0 mg L^−1^), corresponding to 6 of the 7 affected fish (85.7%).

Following exposure to eugenol, 55% of the animals (11/20) presented a period of unresponsiveness during recovery. Among these affected fish, 3 individuals exhibited stereotyped swimming behavior, whereas 8 remained immobile. This altered recovery profile was more frequently observed at the highest eugenol concentration, accounting for 7 of the 11 affected fish (63.6%). Despite a 12 h fasting period, regurgitation of clear gastrointestinal fluid was observed in 20% of the animals (4/20), and one fish presented hyperemia at the base of the fins during recovery. During induction, mild agitation was commonly observed; however, intense agitation occurred exclusively in the eugenol-treated groups, affecting all animals exposed to this anesthetic.

With MS-222, 65% (13/20) of the fish exhibited periods of unresponsiveness during recovery. Additionally, 20% (4/20) exhibited an intermittent recovery pattern, characterized by alternating periods of normal, responsive swimming and immobility associated with a lack of responsiveness to external stimuli. One individual also displayed hyperemia in the fin base region during recovery.

In the statistical analysis, the time for induction and recovery as well as OBR were evaluated. No significant three-way interactions among anesthetic, concentration and sex were observed for any of the analyzed parameters (*p* > 0.05). The only statistically significant effect was the interaction between time and anesthetic type (*p* = 0.004) and between OBR and anesthetic type (*p* > 0.05 across stages). No other main effects or interactions involving concentration, sex, or body weight reached significance (*p* > 0.05 for all).

When evaluating anesthesia stages separately, however, concentration had a significant influence over time to reach stage III and IV of anesthesia (*p* < 0.001) as well as time to regain equilibrium (*p* < 0.007). Analysis of the mean values indicated a concentration-dependent effect, characterized by reduced induction times and prolonged times to equilibrium recovery at higher concentrations. In contrast, the time to full recovery showed different patterns among anesthetics, decreasing with higher concentrations of propofol and MS-222 but increasing at higher eugenol concentrations (Table 1).

The influence of the anesthetic drug was observed on all measured times. Propofol was the first to produce loss of equilibrium; however, it required the longest time to reach stage IV of anesthesia. The opposite pattern was observed with MS-222, which took the longest to produce loss of equilibrium but was the fastest to induce stage IV of anesthesia. Eugenol showed an intermediate profile between these two agents. During recovery, animals anesthetized with MS-222 were the first to resume movement, regain equilibrium, and complete recovery. In contrast, propofol resulted in the longest recovery time, while eugenol maintained an intermediate profile (Figure 1).

Regarding the anesthetic effect on OBR, propofol produced a rapid and pronounced reduction, with significant decreases observed up to anesthesia stage III (*p* = 0.008) and from stage III to stage IV (*p* < 0.011), resulting in the greatest overall reduction (*p* < 0.001). In contrast, MS-222 exhibited a more delayed effect, with no significant differences observed between baseline and stage III anesthesia (*p* = 0.157) and significant differences detected only between stage III and stage IV (*p* = 0.003) and between baseline and stage IV anesthesia (*p* < 0.001). Eugenol only significantly reduced OBR from basal levels to stage IV; however, its effect was less pronounced (*p* = 0.008). After recovery, OBR returned to baseline values in the MS-222 and eugenol groups, while propofol showed a residual effect, with values remaining significantly different from baseline (*p* < 0.001) (Figure 2).

### 3.3. Hematological Analyses

Five fish died during the hematological analysis experiment: one male exposed to the higher concentration of MS-222 and four males exposed to the higher concentration of eugenol. In all cases, fish recovered body movements and opercular beat rate (OBR) after sampling; however, they failed to regain equilibrium and subsequently died before reaching complete recovery. All animals anesthetized with eugenol and propofol remained unconscious after the procedures, while those anesthetized with MS-222 exhibited signs of recovery by the end of blood sampling.

There were no significant differences in erythrogram or leukogram parameters among fish exposed to the different anesthetics (eugenol, propofol, and MS-222) and their respective tested concentrations (*p* > 0.05). Overall, RBC counts averaged 2.12 ± 0.58 × 10^6^ cells·µL^−1^, with HCT and Hb values of 37.42 ± 5.90% and 10.75 ± 3.15 g·dL^−1^, respectively. The erythrocyte indices included an MCV of 186.42 ± 48.89 fL, an MCH of 52.90 ± 17.94 pg·cell^−1^, and an MCHC of 29.06 ± 8.84 g·dL^−1^. Total leukocytes recorded a mean value of 10,108.3 ± 4500.0 cells·µL^−1^. Lymphocytes were the predominant leukocyte population (61.08 ± 13.95%), followed by neutrophils (24.55 ± 10.77%), monocytes (11.02 ± 9.36%), basophils (2.07 ± 1.60%), and eosinophils (0.47 ± 0.79%). No significant difference in plasma glucose or lactate levels was observed among anesthetic agents (*p* > 0.05). However, plasma glucose differed significantly between concentrations of the same anesthetic (*p* = 0.036), with higher concentrations associated with increased glucose levels. In contrast, lactate levels were not influenced by anesthetic concentration (Figure 3) but only differed significantly between weights (*p* = 0.019) and sexes (*p* = 0.006); smaller animals and males showed lower mean values. No other significant interactions among anesthetic type, concentration, body weight, and sex were detected (*p* > 0.05).

Plasma cortisol did not differ significantly (*p* > 0.05) between low and high concentrations of the same anesthetic, although values were consistently lower at high concentrations. Significant differences were observed only in pairwise comparison between the low-concentration propofol group and low-concentration eugenol group (*p* = 0.029). Body weight and sex, as well as the interactions among anesthetic agent, concentration, and sex, did not significantly affect cortisol levels (*p* > 0.05). (Figure 4).

## 4. Discussion

The anesthetic agents evaluated in this study were selected based on their widespread use and well-documented physiological effects in fish under both experimental and aquaculture conditions [42,43]. Eugenol, propofol and MS-222 represent distinct pharmacological classes, allowing a comparative evaluation of anesthetic response profiles. Recent studies involving tropical freshwater and Neotropical fish species have expanded current knowledge regarding anesthetic performance under warm-water conditions; however, direct comparisons between different anesthetic classes in small experimental fish models remain comparatively limited [9]. Moreover, although pairwise comparisons among these anesthetics have been reported, studies directly comparing all three agents under the same experimental framework are still limited, especially in tropical species. A potential limitation of our physiological assessment, however, is the absence of an unhandled baseline control for blood parameters. In small teleosts like *A. lacustris*, conscious blood sampling induces acute handling stress that can confound basal cortisol and glucose levels. Although blood collection under mild sedation is possible, the objective of this study was to evaluate differences among anesthetic treatments rather than to compare physiological responses with a baseline condition. Therefore, blood parameters were evaluated to ensure a standardized comparative baseline across treatments, while OBR provided a non-invasive intra-individual reference measurement for assessing changes associated with anesthesia.

The responses observed in *A. lacustris* indicate that, although all three anesthetics achieved effective induction, they differed in both induction dynamics and recovery patterns, resulting in distinct anesthetic response profiles. Since the preliminary study, different patterns of response have been observed depending on the anesthetic compound and concentration used. Respiratory arrest was the most frequently observed adverse effect, occurring with all anesthetics evaluated. Interestingly, while respiratory arrest was associated with the highest concentrations of MS-222 (150 mg L^−1^) and propofol (7.5 mg L^−1^), it was observed only at the lowest eugenol concentration (30 mg L^−1^).

These findings suggest that the relationship between anesthetic concentration and adverse effects is not always linear [43,44]. During immersion anesthesia, physiological responses may be influenced by several factors, including exposure duration, anesthetic uptake through the gills, individual variability, and the time required to reach the desired anesthetic stage. This pattern suggests that some of these factors may have a greater influence depending on the anesthetic used, resulting in different response patterns among treatments. Additionally, these data highlight an optimal anesthetic concentration range that minimizes undesirable responses, as both excessively high and low concentrations may increase the likelihood of adverse effects.

Propofol induced an early loss of equilibrium, but progression to deep anesthesia was slower when compared to the other two anesthetics. This distinction between early loss of equilibrium (stage III) and progression to deep anesthesia (stage IV) is supported by the stage-specific analysis, indicating that these endpoints are not temporally equivalent across these anesthetics and should be interpreted as distinct components of the induction process. Recovery following propofol exposure was dissociated, characterized by stereotyped swimming and reduced responsiveness. This observation indicates that locomotor activity may return before complete restoration of sensory function and full recovery. The dissociation was more pronounced at the lower anesthetic concentrations, suggesting that concentration may influence the synchronization of motor and sensory recovery, with lower concentrations resulting in a greater temporal discrepancy between the resumption of locomotor activity and complete behavioral recovery. A similar dissociation has been reported in *O. niloticus*, in which propofol increased swimming activity while reducing responsiveness to external stimuli, reflecting fluctuations in sedation depth and incomplete recovery [44]. Comparable alterations in post-anesthetic behavior have also been described in small freshwater fish models exposed to GABAergic anesthetics, indicating that restoration of locomotor activity may not necessarily coincide with complete recovery of responsiveness [45,46].

Eugenol showed a distinct anesthetic profile compared to propofol and MS-222, with marked excitation during induction and a higher frequency of altered recovery patterns. Intense agitation was observed in all animals during induction, in contrast to the milder responses seen with the other anesthetics. Similar excitatory responses during induction have been reported in tropical freshwater species exposed to eugenol, particularly at higher concentrations, indicating that species-specific sensitivity and anesthetic concentration may influence induction dynamics [47]. During recovery, more than half of the fish exhibited unresponsiveness, predominantly associated with immobility, in contrast to the dissociated, active pattern observed with propofol and the more transient alterations seen with MS-222. Higher eugenol concentrations were associated with more pronounced alterations in recovery dynamics and, in contrast to propofol and MS-222, with prolonged times to complete motor and sensory recovery. Similar concentration-dependent alterations have also been described in other fish species, including largemouth bass and Siberian sturgeon [27,48]. In parallel, OBR values progressively decreased with increasing eugenol concentrations, indicating reduced ventilatory activity during anesthetic exposure. Similar ventilatory depression has been reported in fish species exposed to eugenol [47,49], reinforcing the importance of respiratory monitoring during anesthetic induction and recovery.

MS-222 showed a distinct response profile from propofol and eugenol, characterized by faster progression to deep anesthesia and earlier recovery. Notably, the return of responsiveness occurred sooner than with the other anesthetics. However, the post-anesthetic phase was not uniform, as several fish exhibited transient unresponsiveness or intermittent episodes of activity, differing from the dissociated, active pattern observed with propofol and the immobility associated with eugenol. This pattern was more pronounced at the lower anesthetic concentrations and indicates that rapid recovery does not necessarily reflect complete restoration of responsiveness and coordinated activity. Comparable variability has been described in tropical species such as *O. niloticus*, in which MS-222 may alter responsiveness and recovery activity depending on exposure conditions [44]. In contrast, studies in Siberian sturgeon report rapid induction and short recovery times following MS-222 exposure, without specific assessment of behavioral recovery characteristics [48]. Together, these findings suggest that, despite its consistent anesthetic efficiency, MS-222 may produce short-lived but detectable alterations in behavioral recovery, although the extent of these effects appears to vary across species and experimental conditions.

Collectively, these results demonstrate that anesthetic recovery in fish is not solely defined by the return of movement, but also by the restoration of responsiveness and coordinated activity. The dissociation between locomotion and sensory responsiveness observed here, particularly with propofol and MS-222, underscores the importance of incorporating multiple recovery-related endpoints when selecting anesthetic protocols for tropical species. The inclusion of ventilatory parameters such as OBR further reinforces this interpretation, as reduced ventilatory activity persisted in some anesthetic groups despite apparent locomotor recovery.

A small number of deaths occurred during blood sampling procedures, particularly at higher anesthetic concentrations. In small teleost species such as *A. lacustris*, venipuncture and blood withdrawal may represent a substantial physiological challenge, as even relatively small blood losses can represent a considerable proportion of total blood volume. This challenge may be exacerbated under deeper planes of anesthesia, which can impair cardiovascular compensatory mechanisms and reduce the ability of fish to maintain adequate perfusion. Thus, the combination of blood loss associated with sampling and the cardiovascular effects of deeper anesthesia may increase the risk of hemodynamic instability [50,51]. Because no mortality was observed during anesthetic induction or recovery in the absence of blood sampling, blood withdrawal under anesthesia, rather than anesthesia alone, may have contributed to the observed mortality. These findings highlight the importance of carefully balancing anesthetic depth, blood volume collected, procedural invasiveness, and animal welfare when designing experimental protocols involving small fish species.

No significant differences were observed in hematological parameters among the anesthetic agents, which suggests that they equally act upon hematological profiles. This indicates that hematological responses alone may have limited applicability for distinguishing the most suitable anesthetic protocol for fish handling procedures. Although manual cell counting is inherently subject to observer variability and counting errors, which may limit its precision and sensitivity, it remains a widely used and established approach in fish hematology, particularly because the nucleated erythrocytes and thrombocytes of fish can pose challenges for conventional automated hematology analyzers. However, other studies in tropical freshwater fish species exposed to short-term anesthetic protocols also showed hematological parameters remained relatively stable following anesthetic exposure [24].

Similarly, plasma lactate levels did not differ significantly among anesthetic types or concentrations, suggesting that the evaluated anesthetic protocols produced comparable metabolic effects under the conditions of the present study. This likely reflects the fast induction times achieved across all protocols, which prevented prolonged respiratory depression and tissue hypoxia. However, lactate concentration differed significantly according to body weight and sex, suggesting that intrinsic biological factors may influence baseline metabolic responses in *A. lacustris*. This variation may be related to the greater body size and muscle mass of females in this species, as lactate production is closely associated with anaerobic muscular activity. Indeed, larger fish can exhibit greater lactate accumulation in white muscle, reflecting a direct relationship among body size, muscle mass, and lactate production [52]. Consistently, prior research in this species has reported increased plasma lactate concentrations following anesthetic exposure [24]. In the present study, however, lactate concentrations remained comparatively stable across anesthetic treatments. Similar findings have been reported in tropical fish species exposed to short-term anesthetic protocols under controlled handling conditions and brief exposure periods [53].

Plasma glucose levels also did not differ significantly among the anesthetic types tested. However, significant differences were observed across different concentrations of the same anesthetic, with higher concentrations consistently associated with elevated plasma glucose. This pattern suggests that greater anesthetic exposure may trigger greater energy mobilization in *A. lacustris*. Elevated plasma glucose levels under higher eugenol concentration have similarly been reported in juvenile spotted seatrout (*Cynoscion nebulosus*) [54]. Nevertheless, this response is not uniform across the literature, since glycemic dynamics under anesthesia vary substantially depending on the specific compound and species evaluated [53,54].

Previous studies in temperate and cold-water fish species have demonstrated that plasma cortisol responses following anesthetic exposure may vary according to exposure duration, anesthetic concentration, and the pharmacological properties of the compound. In red drum (*Sciaenops ocellatus*), prolonged exposure to MS-222 was associated with increased plasma cortisol concentrations, whereas shorter exposure periods produced different response patterns [1]. Similarly, in Atlantic salmon, exposure to anesthetic agents including MS-222 and isoeugenol was associated with alterations in plasma cortisol concentrations during immersion procedures [8]. In rainbow trout, MS-222 has also been associated with different cortisol profiles compared to alternative anesthetics, further highlighting species-specific and anesthetic-dependent variability in circulating cortisol responses [22].

Taken together, the present findings indicate that no single anesthetic protocol was consistently superior across all evaluated endpoints. Therefore, protocol selection in *A. lacustris* should be guided by the specific objectives of the procedure and the relative importance assigned to different anesthetic characteristics. MS-222 and eugenol may be more suitable for routine procedures, such as vaccination and biometric measurements, due to their faster induction and recovery times. In contrast, the slower progression to deeper anesthetic stages and its apparent safety profile suggest that propofol may be advantageous for procedures requiring prolonged and stable anesthesia, such as blood sampling and minor surgical interventions.

## 5. Conclusions

In conclusion, propofol, eugenol and MS-222 were all effective in inducing anesthesia in *A. lacustris*, each yielding distinct operational and physiological responses. Propofol was characterized by prolonged and dissociated recovery, eugenol induced marked excitation during induction and altered recovery patterns, whereas MS-222 promoted faster induction and earlier recovery of responsiveness, although not consistently accompanied by complete restoration of coordinated activity.

Overall, this study expands current knowledge regarding anesthetic options for Neotropical freshwater species. Our findings reinforce the importance of considering both species-specific responses and procedures when selecting anesthetic protocols for research and routine handling procedures in tropical fish species.

## Figures and Tables

**Figure 1:**
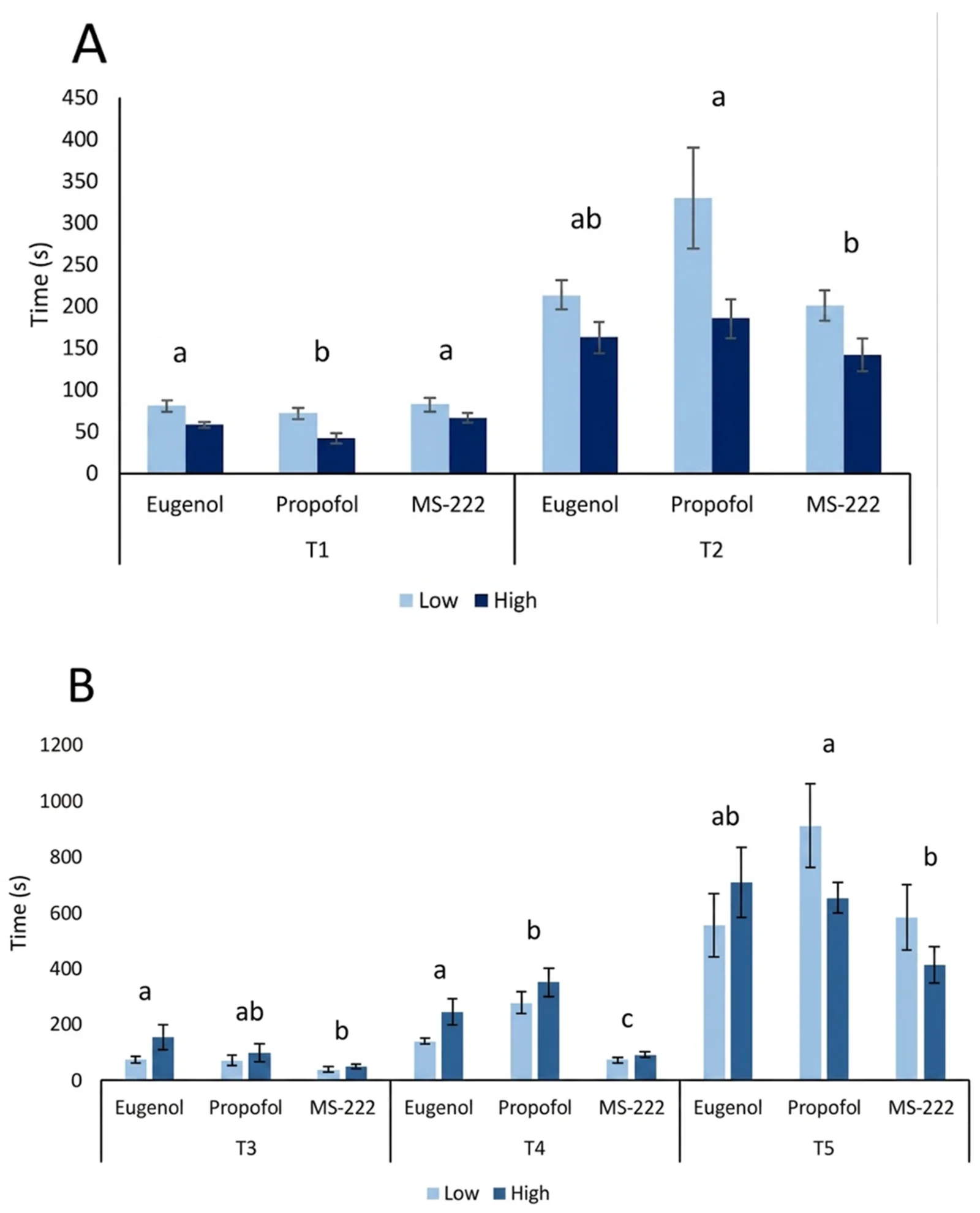
Anesthesia induction and recovery times (s) of *Astyanax lacustris* anesthetized with low and high concentrations of eugenol, MS-222, and propofol. (**A**) Induction phases: stage III (recovery of equilibrium, T1) and stage IV (loss of reflex, T2). (**B**) Recovery phases: beginning of movement (T3), recovery of equilibrium (T4), and full recovery (T5). Data are expressed as means ± SEM (*n* = 10). Weight as covariate (ANCOVA on log10 data). Different letters indicate significance between anesthetics and concentrations within each stage (*p* < 0.05).

**Figure 2:**
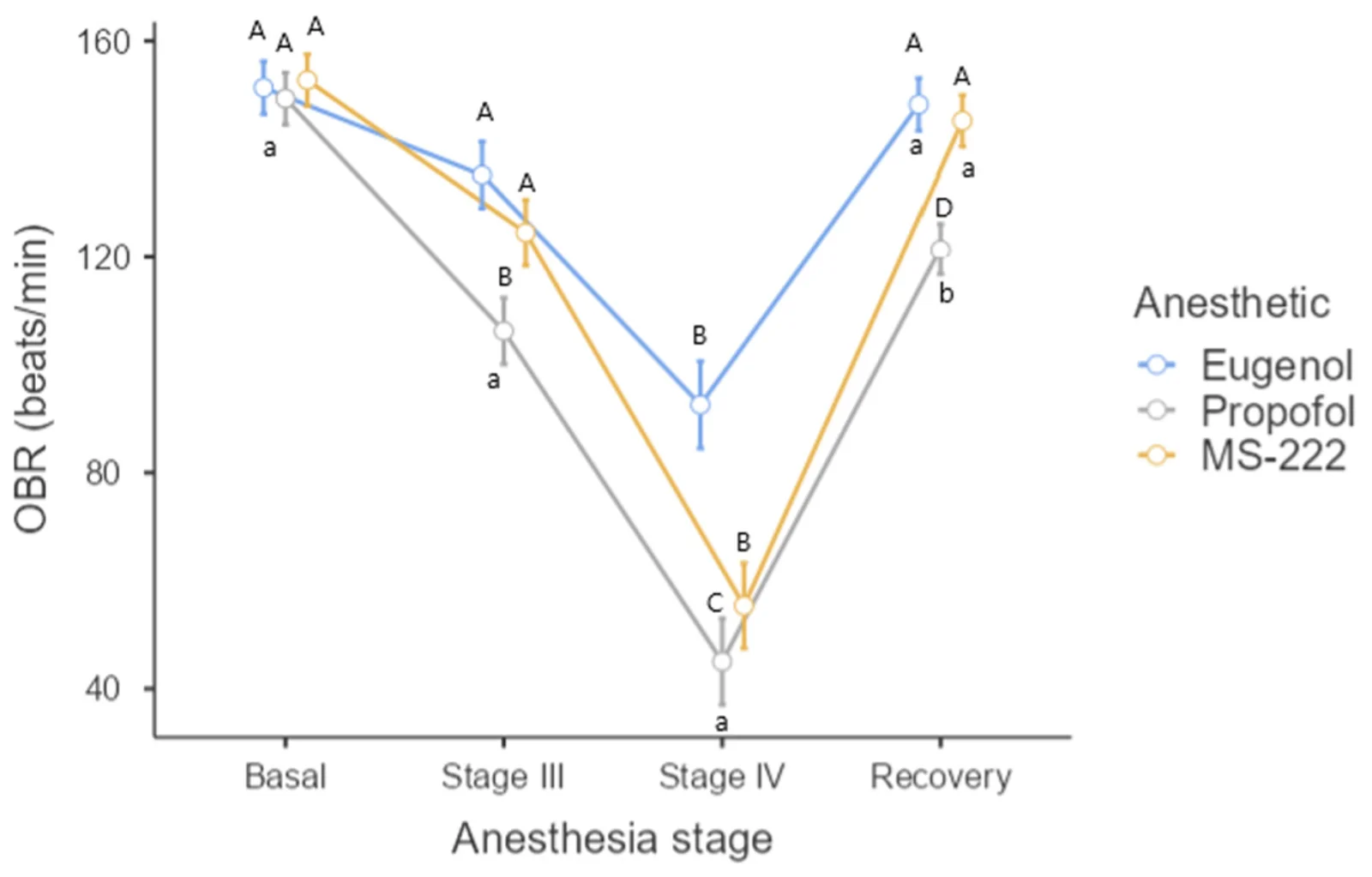
Opercular beat rate OBR (beats/min) of *Astyanax lacustris* during anesthesia. Data are expressed as means ± SEM. Weight as covariate (ANCOVA). Different lowercase letters (between anesthetics) and uppercase letters (between stages) indicate significance (*p* < 0.05).

**Figure 3:**
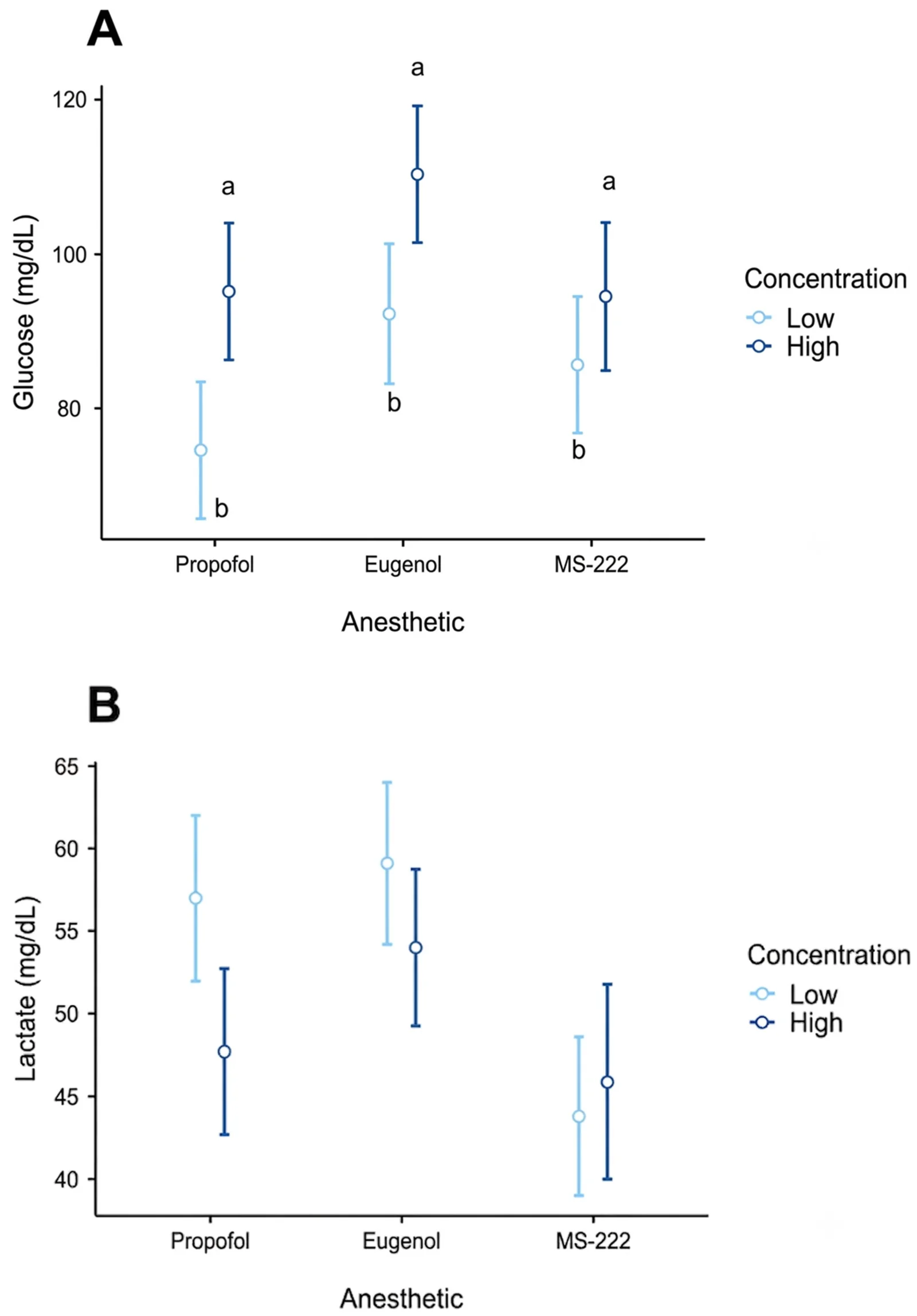
Plasma glucose (**A**) and lactate (**B**) levels in *Astyanax lacustris* subjected to low and high concentrations of propofol, eugenol, and MS-222. Data are expressed as mean ± SEM. Different lowercase letters indicate significant differences between concentrations (*p* < 0.05) according to Tukey’s post hoc test.

**Figure 4:**
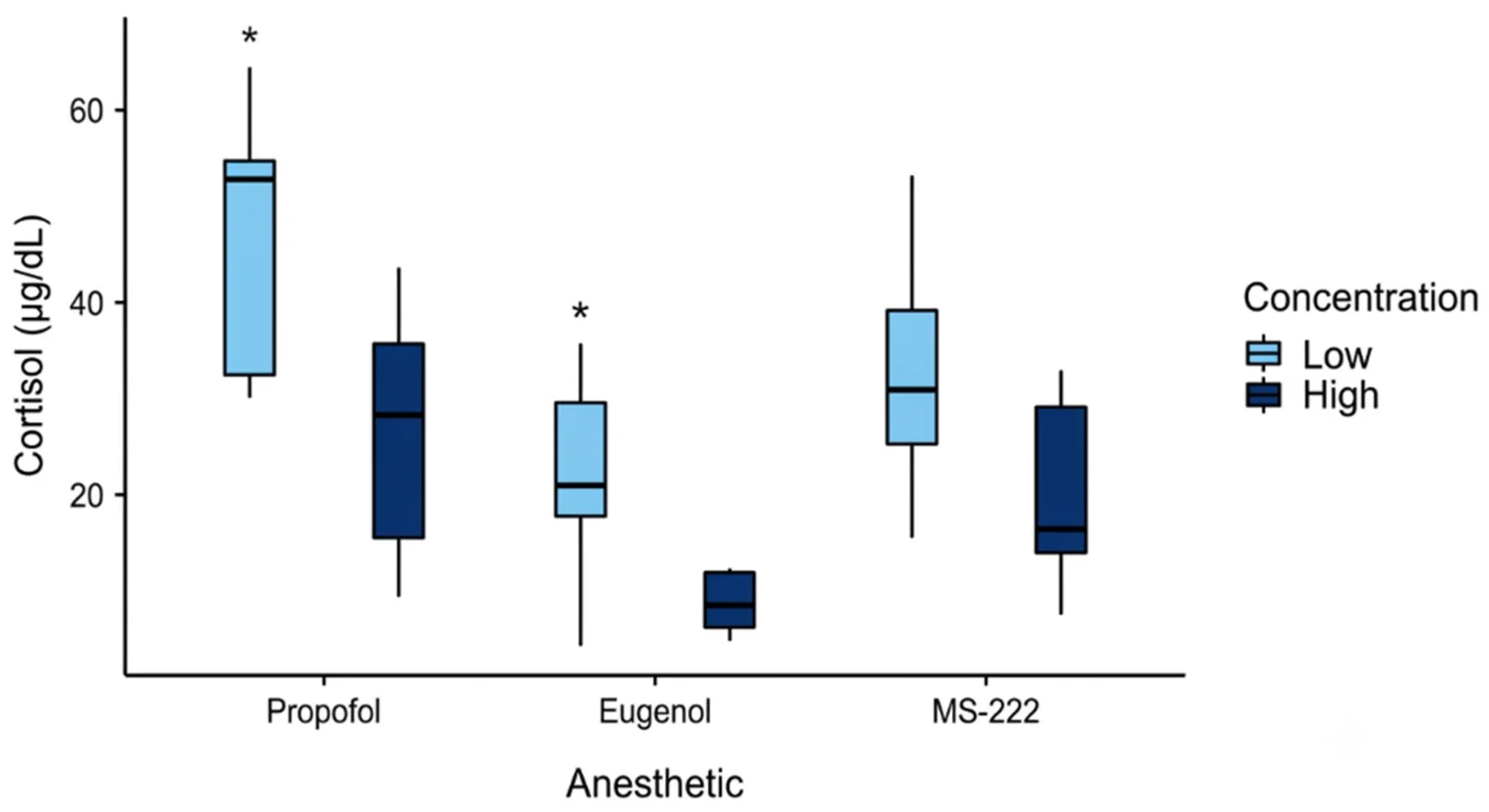
Plasma cortisol levels in *Astyanax lacustris* anesthetized with propofol, eugenol, and MS-222 at low and high concentrations. Boxplots represent the median (line), interquartile range (box), and minimum/maximum values (whiskers). Data are expressed as mean ± SEM. Significant differences between anesthetics according to Tukey’s post hoc test (*p* < 0.05) are denoted by (*).

**Table 1:** Behavioral parameters and opercular beat rate (OBR) in *Astyanax lacustris* under different anesthetic treatments. Values are presented as mean ± SEM (*n* = 10 per group).

			Time (s)					OBR (bpm)			
	Anesthetic	Concentration (mg·L^−1^)	T1 ± SEM	T2 ± SEM	T3 ± SEM	T4 ± SEM	T5 ± SEM	BASAL ± SEM	STAGE III ± SEM	STAGE IV ± SEM	REC ± SEM
Mean	Eugenol	40	80.5 ± 6.3	213 ± 17.2	73 ± 11.2	140 ± 11.9	555 ± 115	145 ± 5.38	137 ± 4.94	103 ± 13.5	145 ± 7.21
		50	58 ± 3.51	162 ± 18.3	155 ± 45.2	244 ± 46.9	709 ± 127	157 ± 7.98	130 ± 4.38	80 ± 14.5	149 ± 6.07
	Propofol	5	71.5 ± 6.67	329 ± 60.5	71.2 ± 17.4	277 ± 39.5	911 ± 151	146 ± 5.88	100 ± 9.01	38 ± 4.7	118 ± 8.85
		6.5	41.7 ± 5.35	185 ± 23.7	97 ± 32.1	351 ± 50.6	654 ± 53.9	153 ± 3.48	115 ± 4.65	53.6 ± 10.2	127 ± 6.82
	MS-222	120	82.5 ± 8.31	201 ± 18.3	38.8 ± 12	71.7 ± 11.5	584 ± 116	145 ± 9.33	126 ± 6.64	48.6 ± 10.8	132 ± 6.14
		130	66.5 ± 5.29	142 ± 19.6	47.5 ± 8.15	90.4 ± 10.1	414 ± 64.8	160 ± 7.29	124 ± 15.3	62.6 ± 8.05	159 ± 6.13
*p*-value anesthetic			0.003 *	0.046 *	0.004 *	<0.001 *	0.014 *	0.017 *			
*p*-value concentration			<0.001 *	<0.001 *	0.076	0.007 *	0.712	0.356			

Induction times: T1, loss of equilibrium; T2, loss of tail-pinch reflex. Recovery times: T3, return of movement; T4, recovery of equilibrium; T5, complete recovery. OBR (beats min^−1^) was measured at baseline, anesthesia stages III and IV, and post-recovery. *p*-values for “anesthetic” and “concentration” represent main effects obtained by ANCOVA (induction and recovery times) and repeated measures ANOVA (OBR). Significant differences (*p* < 0.05) are denoted by (*).

## Data Availability

The data of this manuscript will be available upon request.

## References

[1] Thomas P., Robertson L. (1991). Plasma Cortisol and Glucose Stress Responses of Red Drum (*Sciaenops ocellatus*) to Handling and Shallow Water Stressors and Anesthesia with MS-222, Quinaldine Sulfate and Metomidate. Aquaculture.

[2] Brønstad A. (2022). Good Anesthesia Practice for Fish and Other Aquatics. Biology.

[3] Fayaz I., Qadiri S.S.N., Shah F.A., Dar S.A., Amin A., Mukhtar K., Mandu S.M., Wali A. (2026). Anaesthetics in Aquaculture: Comprehensive Insights into Agents, Mechanisms, and Applications. Fish Physiol. Biochem..

[4] Jia B.-Z., Rui X.-Y., Wang Y., Zeng X., Sheng S.-J., Zeng B.-J., Xu Z.-L., Luo L. (2025). Fishery Anesthetics in Aquaculture Products: Safety Concerns and Analytical Methods. Foods.

[5] Vergneau-Grosset C., Benedetti I.-C.C. (2022). Fish Sedation and Anesthesia. Vet. Clin. N. Am. Exot. Anim. Pract..

[6] Sahinovic M.M., Struys M.M.R.F., Absalom A.R. (2018). Clinical Pharmacokinetics and Pharmacodynamics of Propofol. Clin. Pharmacokinet..

[7] Kiessling A., Johansson D., Zahl I.H., Samuelsen O.B. (2009). Pharmacokinetics, Plasma Cortisol and Effectiveness of Benzocaine, MS-222 and Isoeugenol Measured in Individual Dorsal Aorta-Cannulated Atlantic Salmon (*Salmo salar*) Following Bath Administration. Aquaculture.

[8] Ostrensky A., Pedrazzani A.S., Vicente A.L. (2016). Use of MS-222 (Tricaine Methanesulfonate) and Propofol (2,6-Diisopropylphenol) as Anaesthetics for the Tetra *Astyanax altiparanae* (Teleostei, Characidae). Aquac. Res..

[9] Rairat T., Chi Y., Hsieh C.-Y., Liu Y.-K., Chuchird N., Chou C.-C. (2021). Determination of Optimal Doses and Minimum Effective Concentrations of Tricaine Methanesulfonate, 2-Phenoxyethanol and Eugenol for Laboratory Managements in Nile Tilapia (*Oreochromis niloticus*). Animals.

[10] Sneddon L.U. (2012). Clinical Anesthesia and Analgesia in Fish. J. Exot. Pet Med..

[11] Rairat T., Hsieh C., Thongpiam W., Chou C. (2019). Pharmacokinetic–Pharmacodynamic Modelling for the Determination of Optimal Dosing Regimen of Florfenicol in Nile Tilapia (*Oreochromis niloticus*) at Different Water Temperatures and Antimicrobial Susceptibility Levels. J. Fish Dis..

[12] Corum O., Durna Corum D., Terzi E., Uney K. (2023). Pharmacokinetics, Tissue Residues, and Withdrawal Times of Oxytetracycline in Rainbow Trout (*Oncorhynchus mykiss*) after Single- and Multiple-Dose Oral Administration. Animals.

[13] Yasui G.S., Ferreira do Nascimento N., Pereira-Santos M., Santos Silva A.P.D., Coelho G.C.Z., Visintin J.A., Porto-Foresti F., Okada Nakaghi L.S., Vianna N.C., Carvalho G.B. (2022). Establishing a Model Fish for the Neotropical Region: The Case of the Yellowtail Tetra *Astyanax altiparanae* in Advanced Biotechnology. Front. Genet..

[14] Schleger I.C., Pereira D.M.C., Resende A.C., Romão S., Herrerias T., Neundorf A.K.A., Sloty A.M., Guimarães I.M., De Souza M.R.D.P., Carster G.P. (2022). Cold and Warm Waters: Energy Metabolism and Antioxidant Defenses of the Freshwater Fish *Astyanax lacustris* (Characiformes: Characidae) under Thermal Stress. J. Comp. Physiol. B.

[15] Branco G.S., Cassel M., Chehade C., de Paiva Camargo M., de Melo Dias G.C., Borella M.I., de Jesus L.W.O. (2025). Ontogeny of Adenohypophyseal Cells, Pituitary Gland Development, and Structure in Adults of *Astyanax lacustris* (Teleostei, Characiformes): An Emerging Neotropical Model Fish Species. Fish Physiol. Biochem..

[16] Weinert N.C., Volpato J., Costa Á., Antunes R.R., de Oliveira A.C., Mattoso C.R.S., Saito M.E. (2015). Hematology of Nile Tilapia (*Oreochromis niloticus*) Subjected to Anesthesia and Anticoagulation Protocols. Semin. Ciênc. Agrár..

[17] Nascimento H.D.S., Crispim B.D.A., Francisco L.F.V., Merey F.M., Kummrow F., Viana L.F., Inoue L.A.K.A., Barufatti A. (2020). Genotoxicity Evaluation of Three Anesthetics Commonly Employed in Aquaculture Using *Oreochromis niloticus* and *Astyanax lacustris*. Aquac. Rep..

[18] Grimm K.A., Lamont L.A., Greene S.A., Robertson S., Tranquilli W.J. (2015). Veterinary Anesthesia and Analgesia.

[19] Ohnesorge N., Wilzopolski J., Steinfath M., Lewejohann L., Banneke S., Heinl C. (2024). Assessment of the Effect of Tricaine (MS-222)-Induced Anesthesia on Brain-Wide Neuronal Activity of Zebrafish (*Danio rerio*) Larvae. Front. Neurosci..

[20] Sopinka N.M., Donaldson M., O’Connor C.M., Suski C.D., Cooke S. (2016). Stress Indicators in Fish. Fish Physiology.

[21] Pounder K.C., Mitchell J.L., Thomson J.S., Pottinger T.G., Sneddon L.U. (2018). Physiological and Behavioural Evaluation of Common Anaesthesia Practices in the Rainbow Trout. Appl. Anim. Behav. Sci..

[22] Lemos L.S., Angarica L.M., Hauser-Davis R.A., Quinete N. (2023). Cortisol as a Stress Indicator in Fish: Sampling Methods, Analytical Techniques, and Organic Pollutant Exposure Assessments. Int. J. Environ. Res. Public Health.

[23] Gomulka P., Własow T., Velíšek J., Svobodová Z., Chmielinska E. (2008). Effects of Eugenol and MS-222 Anaesthesia on Siberian Sturgeon *Acipenser baerii* Brandt. Acta Vet. Brno.

[24] Oliveira R.H.F.D., Pereira-da-Silva E.M., Viegas E.M.M. (2019). Clove Oil Attenuates Stress Responses in Lambari, *Astyanax altiparanae*. Aquac. Res..

[25] Oda A., Messenger K.M., Carbajal L., Posner L.P., Gardner B.R., Hammer S.H., Cerreta A.J., Lewbart G.A., Bailey K.M. (2018). Pharmacokinetics and Pharmacodynamic Effects in Koi Carp (*Cyprinus carpio*) Following Immersion in Propofol. Vet. Anaesth. Analg..

[26] Cooke S.J., Suski C.D., Ostrand K.G., Tufts B.L., Wahl D.H. (2004). Behavioral and Physiological Assessment of Low Concentrations of Clove Oil Anaesthetic for Handling and Transporting Largemouth Bass (*Micropterus salmoides*). Aquaculture.

[27] Zahran E., Risha E., Rizk A. (2021). Comparison Propofol and Eugenol Anesthetics Efficacy and Effects on General Health in Nile Tilapia. Aquaculture.

[28] DeTolla L.J., Srinivas S., Whitaker B.R., Andrews C., Hecker B., Kane A.S., Reimschuessel R. (1995). Guidelines for the Care and Use of Fish in Research. ILAR J..

[29] Santiago Rucinque D., Polo G., Borbón J., González Mantilla J.F. (2017). Anesthetic Use of Eugenol and Benzocaine in Juveniles of Red Tilapia (*Oreochromis* Sp.). Rev. Colomb. Cienc. Pecu..

[30] da Paz C.A., da Costa B.M.P.A., Hamoy M.K.O., Dos Santos M.F., da Rocha L.L., da Silva Deiga Y., de Sousa Barbosa A., do Amaral A.L.G., Câmara T.M., Barbosa G.B. (2024). Establishing a Safe Anesthesia Concentration Window for Nile Tilapia (*Oreochromis niloticus*) (Linnaeus 1758) by Monitoring Cardiac Activity in Eugenol Immersion Baths. Comp. Biochem. Physiol. Part C Toxicol. Pharmacol..

[31] Marking L.L., Meyer F.P. (1985). Are Better Anesthetics Needed in Fisheries?. Fisheries.

[32] Canadian Council on Animal Care (2010). CCAC Guidelines on: The Care and Use of Fish in Research, Teaching and Testing.

[33] U.S. Food and Drug Administration (FDA) (2019). FOI Summary Aquacalm.

[34] Hikasa Y., Takase K., Ogasawara T., Ogasawara S. (1986). Anesthesia and Recovery with Tricaine Methanesulfonate, Eugenol and Thiopental Sodium in the Carp, Cyprinus Carpio. Jpn. J. Vet. Sci..

[35] Keene J.L., Noakes D.L.G., Moccia R.D., Soto C.G. (1998). The Efficacy of Clove Oil as an Anaesthetic for Rainbow Trout, *Oncorhynchus mykiss* (Walbaum). Aquac. Res..

[36] Ross L.G., Ross B., Ross B. (2009). Anaesthetic and Sedative Techniques for Aquatic Animals.

[37] Schalm O.W., Jain N.C. (1986). Schalm’s Veterinary Hematology.

[38] Hrubec T.C., Smith S.A., Robertson J.L., Feldman B., Veit H.P., Libey G., Tinker M.K. (1996). Comparison of Hematologic Reference Intervals between Culture System and Type of Hybrid Striped Bass. Am. J. Vet. Res..

[39] de Paiva M.J.T.R., de Pádua S.B., Tavares-Dias M., Egami M.I. (2021). Métodos Para Análise Hematológica em Peixes.

[40] Pickering A.A., Pottinger T. (1983). Seasonal and Diel Changes in Plasma Cortisol Levels of the Brown Trout, *Salmo trutta* L. Gen. Comp. Endocrinol..

[41] Zahl I.H., Kiessling A., Samuelsen O.B., Olsen R.E. (2010). Anesthesia Induces Stress in Atlantic Salmon (*Salmo salar*), Atlantic Cod (*Gadus morhua*) and Atlantic Halibut (*Hippoglossus hippoglossus*). Fish Physiol. Biochem..

[42] Martins T., Valentim A., Pereira N., Antunes L.M. (2019). Anaesthetics and Analgesics Used in Adult Fish for Research: A Review. Lab. Anim..

[43] Félix L., Correia R., Sequeira R., Ribeiro C., Monteiro S., Antunes L., Silva J., Venâncio C., Valentim A. (2021). MS-222 and Propofol Sedation during and after the Simulated Transport of Nile Tilapia (*Oreochromis niloticus*). Biology.

[44] Gressler L.T., Xavier M.L.G., Junior G.B., Loebens L., Barboza V.D.S., Da Costa S.T., Baldisserotto B. (2021). Behavioral and Histological Features of Zebrafish Following Sedation with Eugenol or Propofol. Appl. Anim. Behav. Sci..

[45] Ayala-Soldado N., Mora-Medina R., Molina-López A.M., Lora-Benítez A.J., Moyano-Salvago R. (2024). Evaluation of the Effectiveness of Eugenol and MS-222 as Anesthetics in Zebrafish in Repeated Exposures and Post-Anesthesia Behaviour. Animals.

[46] Simões L.N., Paiva G., Gomes L.D.C. (2010). Óleo de cravo como anestésico em adultos de tilápia-do-nilo. Pesqui. Agropec. Bras..

[47] Noel-Morgan J., Muir W.W. (2018). Anesthesia-Associated Relative Hypovolemia: Mechanisms, Monitoring, and Treatment Considerations. Front. Vet. Sci..

[48] Zang L., Shimada Y., Nishimura Y., Tanaka T., Nishimura N. (2013). A Novel, Reliable Method for Repeated Blood Collection from Aquarium Fish. Zebrafish.

[49] Sousa A.G., Pacheco A.H., Siqueira-Pinto G.A., dos Reis G.T., Fugimura M.M., Vaz L.J., Marcusso P.F., Ramos-Espinoza F.C., da Silva Claudiano G. (2021). Comparative Study of Hematological Parameters of Colossoma Macropomum Anesthetized with Benzocaine and Eugenol by Using Different Anticoagulants. Aquac. Int..

[50] Ferguson R.A., Kieffer J.D., Tufts B.L. (1993). The Effects of Body Size on the Acid–Base and Metabolite Status in the White Muscle of Rainbow Trout Before and After Exhaustive Exercise. J. Exp. Biol..

[51] Jerez-Cepa I., Fernández-Castro M., Del Santo O’Neill T.J., Martos-Sitcha J.A., Martínez-Rodríguez G., Mancera J.M., Ruiz-Jarabo I. (2019). Transport and Recovery of Gilthead Seabream (*Sparus aurata* L.) Sedated with Clove Oil and MS-222: Effects on Stress Axis Regulation and Intermediary Metabolism. Front. Physiol..

[52] Arenas M., Rosales-Arce A., Galan-Conde Y., Sanchés-Fuentes M.G., Barreto A., del Carmen Paredes-Medina A., Maldonado-Flores J.C., Gaxiola G. (2026). Anesthetic Efficacy and Physiological Response to MS-222, Eugenol, and *Cymbopogon flexuosus* Essential Oil in Juvenile Spotted Seatrout (*Cynoscion nebulosus*). Aquac. Res..

[53] Bahi A., Guardiola F.A., Esteban M.A. (2018). A Time Course Study of Glucose Levels and Innate Immune Response in Gilthead Seabream (*Sparus aurata* L.) after Exposure to Clove Oil-Eugenol Derived Anaesthetic. Fish Shellfish Immunol..

[54] Baesso G.M.M., Venâncio A.V., Barca L.C.V., Peppi P.F., Faria C.A., Machado J.P.C., Murgas L.D.S., Bicudo Á.J., de Lima E.M.M., da Cunha Barreto-Vianna A.R. (2024). Exploring the Effects of Eugenol, Menthol, and Lidocaine as Anesthetics on Zebrafish Glucose Homeostasis. Comp. Biochem. Physiol. Part C Toxicol. Pharmacol..

